# Persistent Herpes Simplex Virus Type 1 Infection of Enteric Neurons Triggers CD8^+^ T Cell Response and Gastrointestinal Neuromuscular Dysfunction

**DOI:** 10.3389/fcimb.2021.615350

**Published:** 2021-05-18

**Authors:** Paola Brun, Jessica Conti, Veronica Zatta, Venera Russo, Melania Scarpa, Andromachi Kotsafti, Andrea Porzionato, Raffaele De Caro, Marco Scarpa, Matteo Fassan, Arianna Calistri, Ignazio Castagliuolo

**Affiliations:** ^1^ Department of Molecular Medicine, University of Padova, Padova, Italy; ^2^ Laboratory of Advanced Translational Research, Veneto Institute of Oncology IOV - IRCCS, Padova, Italy; ^3^ Department of Neurosciences, University of Padova, Padova, Italy; ^4^ General Surgery Unit, Azienda Ospedaliera di Padova, Padova, Italy; ^5^ Department of Medicine, Surgical Pathology & Cytopathology Unit, University of Padua, Padua, Italy

**Keywords:** neurotropic virus, neuronal damage, inflammation, intestinal dysmotility, viral replication

## Abstract

Behind the central nervous system, neurotropic viruses can reach and persist even in the enteric nervous system (ENS), the neuronal network embedded in the gut wall. We recently reported that immediately following orogastric (OG) administration, Herpes simplex virus (HSV)-1 infects murine enteric neurons and recruits mononuclear cells in the myenteric plexus. In the current work, we took those findings a step forward by investigating the persistence of HSV-1 in the ENS and the local adaptive immune responses against HSV-1 that might contribute to neuronal damage in an animal model. Our study demonstrated specific viral RNA transcripts and proteins in the longitudinal muscle layer containing the myenteric plexus (LMMP) up to 10 weeks post HSV-1 infection. CD3^+^CD8^+^INFγ^+^ lymphocytes skewed towards HSV-1 antigens infiltrated the myenteric ganglia starting from the 6^th^ week of infection and persist up to 10 weeks post-OG HSV-1 inoculation. CD3^+^CD8^+^ cells isolated from the LMMP of the infected mice recognized HSV-1 antigens expressed by infected enteric neurons. *In vivo*, infiltrating activated lymphocytes were involved in controlling viral replication and intestinal neuromuscular dysfunction. Indeed, by depleting the CD8^+^ cells by administering specific monoclonal antibody we observed a partial amelioration of intestinal dysmotility in HSV-1 infected mice but increased expression of viral genes. Our findings demonstrate that HSV-1 persistently infects enteric neurons that in turn express viral antigens, leading them to recruit activated CD3^+^CD8^+^ lymphocytes. The T-cell responses toward HSV-1 antigens persistently expressed in enteric neurons can alter the integrity of the ENS predisposing to neuromuscular dysfunction.

## Introduction

Herpes simplex virus type 1 (HSV-1) is a successful human pathogen with an estimated seroprevalence of up to 85% in the adult population (Bradley et al., 2014). The primary sites of infection are usually the oral mucoepithelial cells from which HSV-1 spreads to the cell body of sensory neurons located in the trigeminal ganglion to establish a lifelong latent infection (Held and Derfuss, 2011; Swanson and McGavern, 2015). The reactivation of HSV-1 results in the production of infectious viral particles that exit sensory neurons *via* anterograde trafficking and reach the mucoepithelial cells supplied by the nerve. However, compelling evidence indicates a more complex scenario in HSV-1 infection and life cycle. At first, the theory that viral genome replication completely shuts down during HSV-1 latent infection is now being challenged as new evidence indicates that several viral genes such as immediate-early transcripts accumulate in neurons during latency in addition to latency-associated transcripts (Harkness et al., 2014). Secondly, the asymptomatic shedding of HSV-1 from the oral mucosa is higher than previously thought (Kaufman et al., 2005). Finally, infection of peripheral neuronal cells may occur during retrograde axonal transport, implying widespread diffusion of the virus in the host (Koyuncu et al., 2013). Indeed, HSV-1 DNA and HSV-1 latent gene expression have been demonstrated in sensory and autonomic ganglia beyond the trigeminal and in tears, saliva, fecal samples, and nodose ganglia of immunocompetent subjects (Rand et al., 1984; Gesser and Koo, 1997; Gilden et al., 2001; Kaufman et al., 2005). These observations might suggest that HSV-1 can infect the human gastrointestinal tract by swallowing the virus-loaded oral secretions or by neuronal transport. Indeed, several research groups, including our own, have shown that HSV-1 can reach, infect, and persist in the neurons of the enteric nervous system (ENS) in rodents (Gesser and Koo, 1996; Brun et al., 2010; Hill et al., 2012; Brun et al., 2018). In humans, HSV-1 is one of the most important causes of esophagitis in immunocompromised and immunocompetent hosts (McDonald et al., 1985; Mosimann et al., 1994; Rodrigues et al., 2004; Bando et al., 2009; Canalejo et al., 2010; Diezma-Martín et al., 2020). Even if it is not possible to discriminate between reactivation and primary infections, during the past decade, evidence has suggested that the enteric nervous system can be a primary site of reactivation of neurotropic Herpes viruses (Gesser and Koo, 1997; Gilden et al., 2001; Chen et al., 2011; Gershon et al., 2012).

The transient or persistent expression of viral proteins in infected neurons drives strong immune responses that are instrumental in controlling HSV-1 replication (Simmons and Tscharke, 1992; St. Leger and Hendricks, 2011). It has been suggested that infiltrating HSV-1 reactive lymphocytes in the ENS affect the integrity of the enteric neurons in achalasia patients resulting in functional alterations (Facco et al., 2008). Thus, as it does in the central nervous system, HSV-1 might periodically reactivate from latency in the nodose and celiac ganglia (Yoshikawa et al., 1996; Theil et al., 2001; Margolis et al., 2007), thereby keeping the intestinal HSV-1 specific immune response active. Evidence of HSV-1 DNA association with some gastrointestinal lesions such as achalasia and peptic ulcers (Löhr et al., 1989; Facco et al., 2008) supports the hypothesis that gastrointestinal motor disorders characterized by recurrent inflammatory exacerbation are directly or indirectly correlated to HSV-1 infection of the ENS. The mechanisms by which immune system responses damage the enteric neurons during long-term HSV-1 infection are still not well defined.

To better understand the involvement of HSV-1 in the alterations of the enteric neurons, we set up an animal model in which the initial intranasal administration of HSV-1 leads to immunization and latent infection in the brain; the subsequent oral inoculation exposes the ENS to the virus (Brun et al., 2010; Brun et al., 2018). We demonstrated that following the orogastric inoculum, HSV-1 reaches the enteric nerves and establishes infection lasting for at least three weeks (Brun et al., 2018). During the infection, we observed fluctuating viral gene expression levels and significant macrophage infiltration in the ENS directly linked with intestinal dysfunction (Brun et al., 2018). Since in humans gastrointestinal motor disorders are frequently associated with histological findings of infiltrating CD3^+^ lymphocytes (De Giorgio and Camilleri, 2004), the current study was designed to characterize the kinetics of immune cell recruitment in the ENS infected by HSV-1 and to determine the mechanisms by which the virus induces structural and functional anomalies of the myenteric ganglia resulting in gastrointestinal dysmotility.

## Materials and Methods

### Viral Stock Preparation

The HSV-1 strain SC16 was propagated in Vero cells (ATCC^®^ CCL81™, American Type Culture Collection, VA, USA), as described elsewhere (Brun et al., 2010). HSV-1 (1x10^9^ plaque-forming units, PFU/mL) was stored at -80°C. Mock solutions were prepared from Vero cell monolayers not exposed to HSV-1.

### Anti-CD8 Monoclonal Antibody Purification

The anti-CD8 monoclonal antibody was produced by hybridomas in suspension culture in Iscove’s Modified Dulbecco’s Medium containing 10% heat-inactivated fetal bovine serum (FBS; all purchased from Thermo Fisher Scientific). The anti-CD8 antibody was produced by clone 2.13 (ATCC TIB-210). The culture supernatant was collected by centrifugation, diluted 1:1 in Protein Binding Buffer (Thermo Fisher Scientific), and monoclonal antibodies were purified using Protein G PLUS-Agarose column (Santa Cruz Biotechnology; Segrate, Italy). The protein concentration was determined by the Bradford method using commercially available kit (Protein Assay Kit; Bio-Rad Laboratories, Hercules, CA).

### Animal Treatments

Eight-week-old C57/Bl6J male mice purchased from Envigo Company (Udine, Italy) were housed in ventilated rack systems at controlled temperature and humidity with 12-hour light/dark cycles. Regular chow food and tap water were provided *at libitum* throughout the study. Animals were infected with HSV-1 following an infectious protocol that we previously set up and largely described elsewhere (Brun et al., 2018). Briefly, mice were administered by intranasal (IN) route with 1x10^2^ PFU (10 μL final volume) of HSV-1 strain SC16. Four weeks later, mice received 1x10^8^ PFU of HSV-1 in 100 µl final volume *via* the orogastric route (OG). Control mice received equal volumes of Vero cell lysate by intranasal or orogastric route (sham infection). To rule out neuropathological changes and intracerebral viral replication, neurological evaluations were carried out in a dedicated group of HSV-1 infected and sham infected animals. Two investigators in a blinded fashion performed the analyses. Animals were examined twice a week in the early morning for: their spontaneous activity in cage and the ability to approach all four walls of the cage; symmetry in the outstretching of both forelimbs when the animal approach the edge of a table while being held by the tail; ability to climb up the wall of a wire cage using all four limbs and the strength of attachment to the wall when the animal was removed from the wire cage by pulling it off by the tail. The neurological evaluation were scored using a validated system (Garcia et al., 1995). The mice were sacrificed 4, 6, 8, or 10 weeks post OG HSV-1 administration.

For T cell depletion experiments, starting at the 4^th^ week of orogastric infection mice were weekly injected by intraperitoneal (IP) route with 200 μg rat anti-mouse CD8 monoclonal antibody and sacrificed at the 8^th^ week starting from the OG infection. The control mice were treated at the same time points with rat IgG2b isotype antibody (Thermo Fisher Scientific, Monza, Italy).

The animal study was approved by the Animal Care and Use Committee of the University of Padova following the protocols of the Italian Ministry of Health and European guidelines for humane animal use.

### Histological Evaluation

At the time of sacrifice, the gut was removed and specimens of distal ileum from 6-8 mice per experimental group were placed in 10% neutral buffered formalin, embedded in paraffin and sectioned. Sections (5 μm thick) were stained with Haematoxylin and Eosin (H&E). Slides were assessed in a blinded manner. A minimum of 10 independent fields per animal was examined.

### Dissection of Longitudinal Muscle Myenteric Plexus

At the time of sacrifice, the ileum or the colon were washed in oxygenated Krebs solution (126 mM NaCl, 2.5 mM KCl, 25 mM NaHCO_3_, 1.2 mM NaH_2_PO_4_, 1.2 mM MgCl_2_, 2.5 mM CaCl_2_, pH 7.2) and cut in ~1 cm long piece. The longitudinal muscle layers containing the myenteric plexus (LMMP) were peeled off using microdissection forceps as described elsewhere (Brun and Akbarali, 2018). The LMMP were immediately snap-frozen in liquid nitrogen for subsequent nucleic acids or protein extraction. LMMP of the ileum were subjected to enzymatic digestion for the culture of enteric neurons.

### Nucleic Acid Extraction and Analysis

Total RNA was extracted from LMMP using the EZNA lysis buffer (Total RNA Kit I, Omega bio-tek, Italy) and the Retsch MM300 TissueLyser Lab Vibration Mill Mixer (QIAGEN S.r.l., Milan, Italy) (Brun et al., 2013). Contaminating DNA was removed by DNase I treatment (Omega bio-tek). Gene expression was assessed using the iTaq Universal SYBR Green One-Step Kit (Bio-Rad Laboratories; Segrate, Italy); oligonucleotides are listed in **Table 1**. The expression of the targeted mRNA was normalized to 18S ribosomal RNA (*Rn18S*) and plotted as mean fold expression.

**Table 1:** Oligonucleotides and PCR conditions.

gene	sequence	Tm (°C)
LAT	Fw 5’-gtgattctctggctgcaccgcattcttctt-3’ Rv 5’-tgttgggcaggctctggtgttaaccacaga-3’	56
tk	Fw 5’-atcgtctacgtacccgagccgatgacttac-3’ Rv 5’-ggcctggggggtcatgctgcccataaggta-3’	60
ICP0	Fw 5′-ggtgtacctgatagtgggcg-3′ Rv 5′-gctgattgcccgtccagata-3′	60
ICP4	Fw 5′-atgacggggacgagtacgac-3′ Rv 5′-acgacgaggacgaagaggat-3’	56
VP16	Fw 5′-gcccgaatcaacaccataaa-3′ Rv 5′-ccttaacctcccgctggt-3′	60
ICP27	Fw 5′-atgtgcatccaccacaacct-3′ Rv 5′-tccttaatgtccgccagacg -3′	60
ICP47	Fw 5′-tgtttcgcgtgtgtgggacata-3′ Rv 5′-ttgtccttcaggacgggcttc-3′	60
Tk DNA	Fw 5′-tagcccggccgtgtgaca-3′ Rv 5′-cataccggaacgcaccacacaa-3’	60
gB	Fw 5′-ggctccttccgattctcc-3′ Rv 5′-ggtactcggtcaggttggtg-3′	60
gC	Fw 5′-ccaaacccaagaacaacacc-3′ Rv 5′-tgttcgtcaggacctcctct-3′	60
gD	Fw 5′-gccaggttgggggccgtgat-3′ Rv 5′-acctgcggctcgtgaagata-3′	60
*Hbb*	Fw 5’-ggtgaactccgatgaagttg-3’ Rv 5’-gggtaatgccaaagtgaaggcc-3’	60
*Rn18S*	Fw 5′-tcaagaacgaaagtcggagg-3′ Rv 5′-ggacatctaagggcatca-3′	60
*Cxcl 11*	Fw 5′-cagctgctcaaggcttcctta-3′ Rv 5′-ctttgtcgcagccgttactc-3′	60
*Ccl22*	Fw 5′-gcctgctgttcacagttgc-3′ Rv 5′-caggtgagtggggcgtta-3′	60
*Cxcl 10*	Fw 5′-gctgccgtcattttctgc-3′ Rv 5′-tctcactggcccgtcatc-3′	60
*Cxcl9*	Fw 5′-cttttcctcttgggcatcat-3′ Rv 5′-gcatcgtgcattccttatca-3′	60

Fw, forward; Rv, reverse; Tm, melting temperature.

### Western Blot

LMMP were homogenized in RIPA buffer, as detailed elsewhere (Brun et al., 2013). Protein samples (30 µg) were separated using SDS-polyacrylamide gel electrophoresis and then transferred onto nitrocellulose membranes (0.45 µm pore size in roll form; Millipore, Milan, Italy). The membranes were subsequently incubated in 5% w/vol bovine serum albumin (BSA; Merck, Milan, Italy) in TBST (120 mM Tris-HCl pH 7.4, 150 mM NaCl, and 0.05% Tween 20) for 1 hour at room temperature. They were then incubated overnight a 4°C with the appropriate primary antibody (**Table 2**) and, after extensive washing, with the relative horseradish peroxidase-conjugated secondary antibodies (**Table 2**). The immunocomplexes were visualized using enhanced chemiluminescence (Millipore). The images were captured using a Hyper Film MP (GE Healthcare, Milan, Italy). The membranes were probed with anti-β-actin antibody to ensure equal loading.

**Table 2:** Antibodies used in the study.

Primary antibodies			
Antigen (host)	clone	source	application
βIII-tubulin (rabbit), PE conjugate	polyclonal	Millipore	IF, FC
Peripherin (rabbit)	polyclonal	Millipore	IF
S100β (rabbit)	EP1576Y	Millipore	IF
HuC/HuD, biotin conjugate	16A11	Invitrogen	IF
nNOS (rabbit)	polyclonal	Invitrogen	IF
βIII-tubulin (mouse)	TU-20	Millipore	WB
GFAP, PE conjugate	GA-5	Millipore	FC
αSMA, PE conjugate	1A4	Merck	FC
pan HSV1 (rabbit), FITC conjugate	polyclonal	GeneTex	FC
pan HSV1+HSV-2 (rabbit)	polyclonal	Dako	IHC
HSV1/2 gD (mouse)	H170	Santa Cruz Biotech	WB
HSV1/2 gB (mouse)	10B7	Santa Cruz Biotech	WB
CD69 (mouse) PE conjugate	H1.2F3	eBioscience	FC
β-actin (mouse)	AC-15	Sigma-Aldrich	WB
CD3 (rat)	17A2	eBioscience	IHC, FC
CD4 (rabbit) Cy7 conjugate	50134-R001	Sino Biological Inc	FC
CD8 (mouse) FITC conjugate	53-6.7	Invitrogen	FC
CD8 (rat) PE conjugate	76-2-11	Invitrogen	FC
CD8 (rat) Cy7 conjugate	5H10	Invitrogen	FC
IFNγ (mouse) FITC conjugate	XMG1.2	eBioscience	FC
F4/80 (rat)	CI:A3-1	Abcam	FC
CD11c (mouse) Cy7 conjugate	N418	eBioscience	FC
CD19 (rabbit) FITC conjugate	C1C3	Gene Tex	FC
NK1.1 (rabbit) FITC conjugate	PK136	Gene Tex	FC
VP16 (rabbit)	polyclonal	GeneTex	WB
IL17 (rabbit) FITC conjugate	TC11-8H4	Molecular probes	FC
Perforin (rat) FITC conjugate	eBioOMAK-D	eBioscience	FC
Granzyme B (rat) FITC conjugate	NGZB	eBioscience	FC

Secondary antibodies		
Antigen (host)	source	application
anti-rabbit (goat) PE	Chemicon	IF
anti-rat (rabbit) FITC	Invitrogen	FC,
anti-rabbit (goat) HRP	Sigma-Aldrich	WB, IHC
anti-mouse (goat) HRP	Sigma-Aldrich	WB
anti-rat (rabbit) HRP	Sigma-Aldrich	IHC
anti-rabbit (goat) APC	Chemicon	FC
anti-rat (rabbit) PE	Sigma-Aldrich	FC
anti-rat (rabbit) Cy7	Invitrogen	FC
Streptavidin Alexa Fluor 488 conjugate	Invitrogen	IF

WB, western blot; IHC, immunohistochemistry; FC, flow cytometry; IF, immunofluorescence.

### Isolation, Culture, and Immunofluorescence Analysis of Enteric Neurons

The enteric neurons were cultured from LMMP preparations (see *Dissection of Longitudinal Muscle Myenteric Plexus* section) obtained under sterile conditions. LMMP were immediately transferred to an ice-cold oxygenated Krebs solution and subjected to enzymatic dissociation using collagenase type II as previously detailed (Brun and Akbarali, 2018; Brun et al., 2019). The cells were resuspended in Neurobasal A media containing B-27 supplement, 1% FBS, 10 ng/mL nerve growth factor, penicillin/streptomycin (1% vol/vol) (all provided by Thermo Fisher), and acyclovir (100 μM; Merck). Cell suspensions were seeded on laminin and poly-D-lysine (all purchased from Merck) pre-coated coverslips and culture for ten days. Media were renewed every two days.

Acyclovir was removed on the fifth or ninth day of culture. Cells were then co-cultured with lymphocytes or fixed with buffered PFA 4% for 10 minutes and subjected to immunofluorescence using anti-HSV-1 and anti-βIII-tubulin antibodies (**Table 2**). The coverslips were mounted using a Prolong Antifade kit, and they were analysed using a Leica TCSNT/SP2 confocal microscope (Leica Microsystems, Wetzlar, Germany).

### Immunohistochemistry

Immunohistochemical analyses (IHC) were performed on formalin-fixed, paraffin-embedded ileal samples. Sections were deparaffinized, rehydrated, and treated with 10 mmol/L sodium citrate buffer pH 6.0 (90°C, 20 min). Sections were incubated with 0.3% H_2_O_2_ and with blocking serum (0.04% bovine serum albumin, 0.5% normal goat serum) for 30 minutes, and then with the appropriate primary antibody (**Table 2**) for one hour at 22°C. After washing in PBS, preparations were exposed for 30 minutes to secondary antibody (**Table 2**). The immunocomplexes were uncovered using Dako Envision^+^ System-HRP labeled Polymer Detection system (Dako, USA). Finally, the sections were counterstained with H&E and mounted. Sections incubated with isotype-matched antibody served as negative controls. The images were captured using a Leica DM4500B microscope equipped with a high-resolution camera.

### Ileal Whole Mount Preparation and Staining

Whole mount preparations of the ileum were obtained as previously describe (Brun et al., 2013). Briefly, 8 cm segment of the distal ileum was flashed with PBS, filled with 4% PFA and submerged in the same fixative for 1 h at 22°C. Tissues were then washed in PBS (3x10min) and stored at 4°C. Whole mounts were prepared from 1 cm long specimen under a dissecting microscope (Zeiss, Germany) by peeling off the LMMP. Tissue was gently stretched, pinned down on a wax support, washed twice with PBS containing 0.5% Triton-X100, incubated in blocking buffer (2% bovine serum albumin, 0.5% Triton-X100 in PBS) and stained at 4°C for 16 h with primary antibody (**Table 2**). After washing in PBS (3x10 min), samples were incubated with fluorescent labelled secondary antibodies (1 h at 22°C, **Table 2**). Images were capture using Leica TCSNT/SP2 confocal microscope (Leica Microsystems).

### Gastrointestinal Transit

*In vivo* gastrointestinal motility was assessed by administering the mice with 70 µl of fluorescein-isothiocyanate dextran (70.000 MW; MP Biomedicals LLC, DBA Italia, Segrate, Italy). Dextran was dissolved in PBS (6.25 mg/mL) and administered to mice *via* oral gavage with minimum animal handling. In line with our set up experiments (Brun et al., 2013), the fluorescent probe in faecal samples was evaluated in animals sacrificed 60 min after probe administration. The stomach, caecum, and colon were examined separately. The small intestine was cut into 8 identical segments. The luminal contents of each segment were collected and centrifuged (10,000 x*g*, 15 min, 4°C). Fluorescence analysis was performed at 494/521 nm (Hitachi-F2000). Gastric emptying was quantified by measuring the percentage of the fluorescent probe that emptied the stomach. The gastrointestinal transit was evaluated by calculating the geometric centre (GC) of distribution of the fluorescent probe.

### Colonic Motility

Mice fasted for 16 h were lightly anesthetized with isoflurane (Merial, France) and a 2-mm glass bead was inserted into the distal colon at 2 cm from the anus. Mice readily regained consciousness and were placed individually in cage. Distal colonic motility was determined by monitoring the time required for the expulsion of the glass bead and reported as the time (seconds) of bead retention (Anitha et al., 2016). In a separate set of experiments, non-anesthetized mice were placed in clean cages and the number of pellets expelled over an hour was determined. To ensure reproducibility among the different time points, gastrointestinal transit (see *Gastrointestinal Transit* section) and colonic motility assays were always performed in the early morning hours (between 8 and 10 in the morning).

### Flow Cytometry

Freshly obtained LMMP preparations (see *Dissection of Longitudinal Muscle Myenteric Plexus* section) were incubated for 10 min at 37°C with collagenase type II from *Clostridium histolyticum* (10 mg/ml), dispase (60 μg/ml) and DNase I (10 μg/mL, all purchased from Merck). Tissue debris were filtered through cell strainers and cells were stained for flow cytometry analysis. Alternatively, cells were purified by density gradient centrifugation through Ficoll-Hypaque (20 minutes at 600x*g* at room temperature; Merck). Mononuclear cells (10^7^ cells/mL) were:

- incubated for 30 min in ice-cold PBS containing 2% BSA or murine serum (blocking buffer) to block unspecific bindings. Cells were washed by centrifugation in ice cold PBS (1600 rpm, 6 min, twice) and incubated with the appropriate antibodies (**Table 2**) diluted in blocking buffer. For intracellular cytokine staining, cells were subsequently incubated for 30 min at room temperature in fixation/permeabilization buffer (eBioscience; Milan, Italy) and then for 60 min at room temperature with the appropriate antibody (**Table 2**) diluted in fixation/permeabilization buffer. Fluorescent signals were recorded using BD-FACSCalibur or BD-FACSCanto II (Becton Dickinson) using CellQuest software (Becton Dickinson). The results were analyzed using the WinMDI or BD FlowJo softwares
- mononuclear cells isolated from LMMP preparations obtained from 8 weeks HSV-1 infected and sham infected mice were stained with anti-CD3 antibody; FITC-conjugated anti-CD8α antibody (clone 53-6.7, Invitrogen); PE-conjugated MHC class I tetramer complexed with the gB (LMWYELSKI) peptide (immunAware Aps, Denmark). Staining was performed in 2% BSA for 60 min at 4°C. Cells were washed and analyzed by flow cytometry, as described above
- cultured (18 h at 37°C) in RMPI 1640 medium supplemented with 10% FBS and 1% penicillin/streptomycin in the presence or absence of UV-inactivated HSV-1 (MOI 1:1). To inhibit protein transport from the endoplasmic reticulum to the Golgi complex and hence increase protein concentration in the cytoplasm, brefeldin A was added (1 µg/mL, GolgiPlug, BD Biosciences; Milan, Italy) for the last 4 hrs of culture. The cells were then collected, stained, and analyzed for intracellular cytokine staining by flow cytometer, as described above
- incubated with CD8 microbeads conjugated antibody (MACS^®^ Microbeads, Miltenyi Biotec; Bologna, Italy) for 30 min in ice. After washing in ice cold PBS (1600 rpm, 6 min, twice), CD8^+^ cells were magnetically separated by positive selection using MACS^®^ Columns (Miltenyi Biotec). Cells were counted using trypan blue exclusion method and 1x10^6^ cells were co-incubated with cultured enteric neurons obtained as described in *Isolation, Culture, and Immunofluorescence Analysis of Enteric Neurons*. Twenty-four hours later, the lymphocytes were collected and subjected to flow cytometry analysis, as described above.

### Statistical Analysis

The data are reported as mean values ± standard error of the mean (SEM). Statistical analysis was performed using GraphPad Prism 7 software (GraphPad, San Diego, CA, United States). Statistical differences were assessed by one-way ANOVA and Bonferroni multicomparison *post hoc* test. Statistical significance was considered for *p* values of 0.05 or less. Number of experiments and replications and statistical significance are reported in the legends of the figures.

## Results

### Orogastric Inoculum Leads to HSV-1 Persistent Infection of the Enteric Nervous System

In the human host, HSV-1 establishes a life-long latent infection within sensory neurons of the trigeminal ganglia (Held and Derfuss, 2011). As HSV-1 has been found in human saliva, our hypothesis is that following recurrent reactivation in the oropharyngeal mucosa, HSV-1 particles may be swallowed and reach the gastrointestinal tract. Indeed, viral DNA was retrieved in faecal samples and enteric ganglia (Rand et al., 1984; Gesser and Koo, 1997), whereas HSV-1 reactive lymphocytes have been reported to infiltrate the oesophageal wall (Facco et al., 2008). We set up a protocol of infection in mice in which animals are inoculated with HSV-1 first by intranasal (IN) installation and then by orogastric (OG) administration. Indeed, previous studies reported that IN inoculum of HSV-1 results in asymptomatic infection in the brain of mice (Boggian et al., 2000). Next, we confirmed that the IN inoculum of low HSV-1 load (1x10^2^ PFU) establishes latent infection in the brain and primes the systemic immune response to protect the animals against the second OG viral inoculum (Brun et al., 2010). We recently reported that HSV-1 persists in the murine ENS for up to 3 weeks following OG inoculum (Brun et al., 2018).

**Figure 1:**
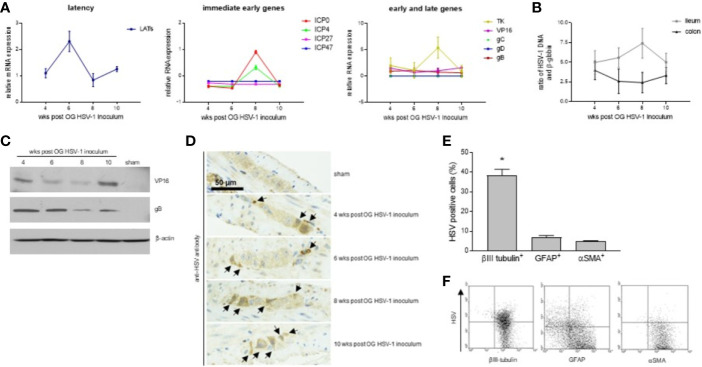
HSV-1 infection of the ENS. **(A)** Total RNA was extracted from the LMMP of mice at 4, 6, 8, or 10 weeks (wks) post orogastric (OG) HSV-1 inoculum. Quantitative PCR were performed to evaluate the expression of latency associated transcripts (LATs), immediate early, early, and late HSV-1 genes. The mean ± SEM of the expression levels of viral genes relative to those of the *Rn18S* housekeeping gene is reported. **(B)** DNA was extracted from the LMMP of ileum and colon of mice at 4, 6, 8, or 10 weeks (wks) post orogastric (OG) HSV-1 inoculum. Quantitative PCR was performed to evaluate the HSV-1 tk and β-globin. Data are reported as the mean ± SEM of the ratio between HSV-1 tk and β-globin (*Hbb*). Experiments described in **(A, B)** were repeated 3 times; n=6-8 mice per experimental group. **(C)** The expression of the HSV-1 proteins was assessed by Western blot analysis of extracts from the LMMP of the ileum of sham- and HSV-1-infected mice. β-actin served as the control loading. Representative images of 4 independent experiments; n=1 mice per experimental group. **(D)** Immunohistochemistry was performed using the anti-HSV pan antibody on ileum sections of the sham- and HSV-1-infected animals. Black arrows indicate positive cells. Representative images of four independent experiments. Scale bars: 50 µm. Representative images of 3 independent experiments; n=3 mice per experimental group. **(E)** LMMP preparations were obtained from the ileum of 8 wks HSV-1-infected mice. The samples were enzymatically digested and the resulting cell suspensions were labeled with pan HSV FITC-conjugated polyclonal antibody and PE-conjugated βIII-tubulin (to detect neuronal cells), PE-conjugated GFAP (glial cells), or PE-conjugated αSMA (muscle cells). Samples were analyzed by flow cytometry. Cells were first selected on forward and side scatter dot plot; then double positive cells were collected in 10,000 events. Data are reported as the percentage (mean ± SEM) of 3 independent experiments; n=3 mice per experimental group. * indicates *p*<0.05 *vs* GFAP-HSV and αSMA-HSV double positive cells. **(F)** Representative dot plots of analysis described in **(E)**.

In this study, we decided to take our investigation a step further by examining HSV-1 infection of the ENS for a longer period, namely 4-10 weeks following viral OG administration. Although at fluctuating levels, RNA of immediate early (ICP4, ICP0, ICP27, and ICP47), early (thymidine kinase, tk), and late genes (VP16 and gB) were detected in the LMMP isolated from the small intestine up to 10 weeks after OG administration (**Figure 1A**). RNA of gC and gD were not detected in the LMMP of the mice sacrificed at 4, 6, 8, or 10 weeks post OG HSV-1 inoculation (**Figure 1A**). Transcripts associated with the latency were also detected (**Figure 1A**). HSV-1 DNA was detected in the LMMP of the ileum and colon in all the samples with no significant variations among the different analyzed experimental times (**Figure 1B**). We confirmed the expression of VP-16 and gB proteins by Western Blot analysis in the LMMP (**Figure 1C**). A specific anti-gD antibody failed to demonstrate the expression of the glycoprotein in the same protein lysates (n=4 for each time point, data not shown).

By IHC, HSV-1 antigens were demonstrated in the ileal myenteric ganglia of the OG-infected but not of the sham-infected mice (**Figure 1D**). To better characterize the cell populations infected by HSV-1 in the myenteric ganglia, LMMP preparations from mice sacrificed at 8 weeks post OG infection (the time at which HSV-1 gene expressions were at the maximum levels, **Figures 1A, B**) were subjected to enzymatic digestion. Cells were stained with βIII-tubulin to detect neuronal cells, GFAP to detect glial cells, αSMA to detect muscle cells, and pan HSV1+HSV2 FITC-conjugated polyclonal antibody. As reported in **Figures 1E, F**, HSV antigens co-localized with βIII-tubulin expression; few GFAP^+^ and αSMA^+^ cells reported fluorescence for HSV antigens. Overall, our results demonstrate that following OG inoculum, HSV-1 infects the neuronal cells in the myenteric plexus of mice.

### Persistent HSV-1 Infection of the ENS Causes Fluctuating Episodes of Gastrointestinal Dysmotility

**Figure 2:**
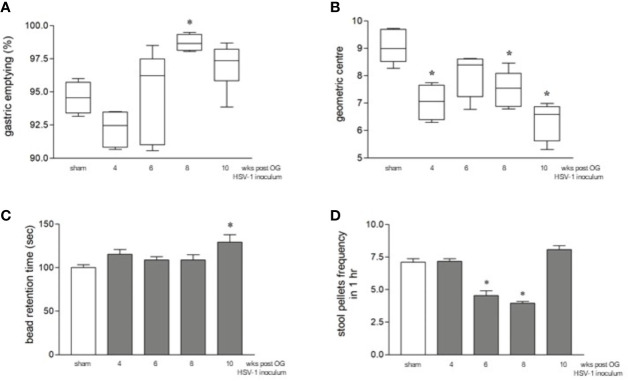
Gastrointestinal dysmotility in HSV-1 infected mice. **(A)** The mice were OG dosed with non-absorbable FITC-labeled dextran and sacrificed 60 minutes later. Gastric emptying was calculated as the percentage of dextran retained in the stomach with respect to the total amount of fluorescence in the gastrointestinal tract. * denotes *p* < 0.01 *vs* sham infected mice. **(B)** Intestinal transit refers to the geometric center that is the center of the distribution of fluorescent dextran in the ileum. **(C)** The time (seconds, sec) required for the expulsion of a glass bead inserted into the rectum. * denotes *p* < 0.05 *vs* sham-infected mice **(D)** The number of fecal pellets expelled in 1 hour (hr). * denotes *p* < 0.05 *vs* the sham-infected mice. Data are reported as mean ± SEM. All the experiments were repeated 3 times; n=6-12 mice per group.

Although the orogastric administration of HSV-1 did not cause neurological deficits (data not shown) or intestinal histologic damage (**Figure S1**) up to ten weeks post-infection, a time-dependent intestinal dysmotility was observed. Sham-infected mice were sacrificed at the same time points as HSV-1 infected mice. Since data relative to intestinal motility obtained from sham-infected mice were comparable among the different times of experiments (**Figures S2A, B**), we decided to pool the data to create a single sham-infected group. Compared with this sham-infected group, 8 weeks after OG HSV-1 administration, the infected mice showed faster gastric emptying (**Figure 2A**). Inversely, slower intestinal transit was observed throughout our experiments in the HSV-1 infected mice. Delayed intestinal transit was more evident in mice sacrificed at the 4^th^, 8^th^, and 10^th^ week post OG inoculum (*p*<0.05 *vs* sham infected mice, **Figure 2B**).

We also observed impaired colonic motility as reported by the longer retention times of the rectal beads in mice at the 10^th^ week of infection (*p*<0.05 *vs* sham-infected mice) and a lower number of fecal pellets expelled over one hour in mice at the 6^th^ and 8^th^ week of infection (*p*<0.05 *vs* sham-infected mice, **Figures 2C, D**). Beads retention time is a marker of rapid propulsion, a colonic movement required only during defecation that usually occurs only when a mass is present in the colon. The significant increase in the frequency of mass movements observed at the 10^th^ week of infection (**Figure 2C**) indicates faster colonic movements (Sarna, 2004). On the contrary, the reduced frequency of fecal pellets in mice at the 6^th^ and 8^th^ weeks of infection (**Figure 2D**) indicates alterations in colonic mucosal secretion and liquid adsorption (Sarna, 2004).

Therefore, HSV-1 infection of the myenteric plexus (**Figure 1**) leads to impaired intestinal motility. Several experimental and clinical observations reported that during infection and inflammation, the plasticity of the ENS induces non-uniform processing of the neurochemical code that changes the structure and function of the neuronal populations (Brierley and Linden, 2014; Lomax et al., 2017; Grabauskas and Owyang, 2017). These observations could in part explain the different and no concordant alterations in motility that we observed in the stomach, the ileum, and the colon in HSV-1 infected mice or the alternating relaxation and contraction along the gastrointestinal tract. Indeed, the plasticity of the ENS explains the different contractive responses observed in the stomach or ileum in *Schistosma* spp. infected mice (Moreels et al., 2001). In humans, the effects of neuroinflammation persist for weeks and extend beyond the point at which the damage was detected (Mawe et al., 2009). Thus, patients suffering from inflammatory bowel diseases report alternating constipation and diarrhoea, whereas patients in remission from Crohn’s disease exhibit delayed gastric emptying (Hungin et al., 2005; Nóbrega et al., 2012).

### HSV-1 Infection Induces Structural Changes of the ENS

Since the enteric glia and neuronal cells control intestinal motility and epithelial barrier function (Brierley and Linden, 2014; Sharkey, 2015; Yoo and Mazmanian, 2017), the loss of coordinated intestinal motility observed in **Figure 2** prompted us to evaluate the integrity of the neuroglial network in the gut wall.

**Figure 3:**
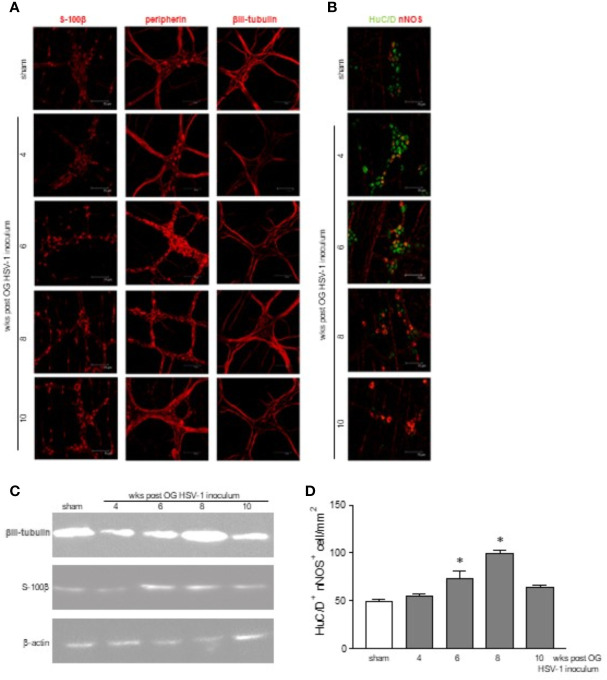
Structural abnormalities in the myenteric plexus during HSV-1 infection. **(A)** Immunofluorescence on whole mount preparations of distal ileum for S-100β (glial marker), peripherin and βIII-tubulin (neuronal markers) was performed. Representative images of 4 independent experiments; n=1 mouse per experimental group; 10 independent fields per animal were examined. Scale bars: 75 µm. **(B)** Immunofluorescence on whole mount preparations of distal ileum for HuC/D (neuronal marker, in green) and nNOS (in red). Representative images of 6 independent experiments. n=1 mice per experimental group; 10 independent fields per animal were examined. Scale bars: 75 µm. **(C)** Western blot analysis of S-100β expression on protein extracts obtained from LMMP of sham and HSV-1 infected mice. β-actin was used as loading control. Representative images of 3 independent experiments are reported. **(D)** HuC/D^+^ nNOS^+^ cells analyzed as in **(B)** were enumerated as described in Results. Two investigators performed blinded image analyses. Data are reported as mean ± SEM of the number of positive cells/1mm^2^. * denotes *p* < 0.05 *vs* sham infected mice.

The immunofluorescence performed in the whole mount preparations of the ileum reported time-dependent abnormalities in the neurons. Fragmented peripherin-related signals were evident at the 6^th^ and 8^th^ week post viral inoculation in the infected mice, whereas βIII-tubulin immunoreactivity was reduced at the 4^th^ week but increased at the 8^th^ week post-IG HSV-1 exposure (**Figure 3A**). Moreover, we uncovered an enhanced expression of the S-100β glial marker 6, 8, and 10 weeks post infection (**Figure 3A**), suggesting that the enteric glial cells were activated (Sharkey, 2015). Altered expressions of βIII-tubulin and S-100β were confirmed by Western blot analysis (**Figure 3C**).

HSV-1 exposure modified the neurochemical code in the myenteric plexus as demonstrated by the altered expression of nNOS in βIII-tubulin cells (**Figure 3B**). HuC/D^+^ nNOS^+^ cells were enumerated in a minimum of 6 randomly selected fields covering 1.254 mm^2^ from at least 6 different whole mount preparations per each experimental group. As reported in **Figure 3D**, a significant increase in HuC/D^+^ nNOS^+^ neurons was reported at the 6^th^ and 8^th^ weeks of infection.

### CD3^+^ T Cells Infiltrate the Myenteric Ganglia During Persistent HSV-1 Infection

T lymphocytes survey the latently infected neurons to prevent reactivation during HSV-1 infection whereas cytokines released by HSV-1 activated lymphocytes have been suggested to contribute to neuronal damage and functional alterations (Egan et al., 2013).

**Figure 4:**
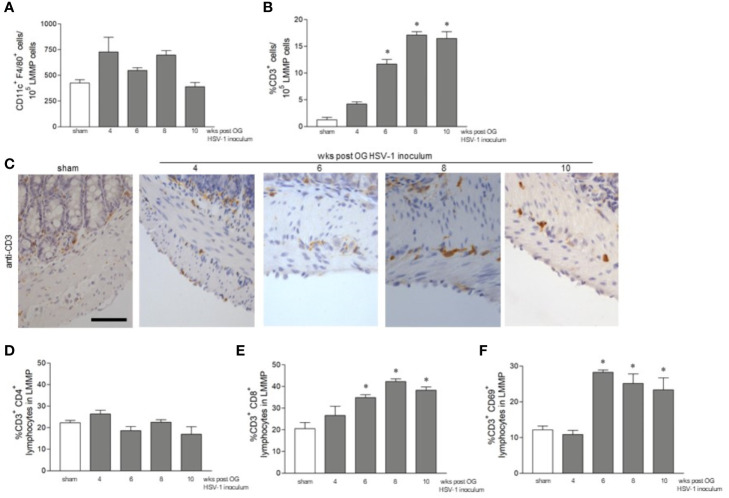
CD3^+^ cells infiltrate the LMMP following HSV-1 infection. **(A)** LMMP preparations were obtained from the ileum of sham- and HSV-1-infected mice as described in Methods. The samples were enzymatically digested, and the resulting cell suspensions were labeled with anti-CD11c and anti-F4/80 antibodies and analyzed using flow cytometry. Cells were first selected on a forward scatter and side scatter dot plot and then double positive cells were recorded. The data are reported as the number of CD11c^+^ F4/80^+^ cells detected in 10^5^ events. The experiments were repeated 2 times; n=4 mice per group. **(B)** Cell suspension obtained as described in **(A)** were labeled with anti-CD3 antibody and analyzed by flow cytometry. Data are reported as the percentage of CD3^+^ cells in 10^5^ events on side scatter dot plot. The experiments were repeated 2 times; n=4 mice per group. * denotes *p* < 0.05 *vs* sham infected mice. **(C)** Sections of ileum obtained from the sham or HSV-1 infected mice were subjected to immunohistochemistry using anti-CD3 antibody. Scale bars: 50 μm. Representative images of four separate experiments; n=3 mice per group. **(D)** Cell suspensions obtained from the LMMP, as described in **(A)**, were labeled with anti-CD3 and anti-CD4 antibodies **(E)** or with anti-CD3 and anti-CD8 antibodies **(F)** or with anti-CD3 and anti-CD69 antibodies. The samples were analyzed using flow cytometry. Cells were first selected on a forward scatter and side scatter dot plot and then double positive cells were recorded in 10,000 events. Data are reported as the percentage of double positive cells. Experiments were repeated 3 times n=4 mice per group. * denotes *p *< 0.05 *vs* sham infected mice.

We set out to investigate the features of the adaptive immune response during persistent ENS infection with HSV-1. Mononuclear cells infiltrating the LMMP were isolated and CD11c^+^F4/80^+^ phagocytes, CD3^+^ T lymphocytes, CD3^-^CD19^+^ B lymphocytes, and CD3^-^NK1.1^+^ NK T cells were characterized by flow cytometry analysis. Compared with the sham infected animals, the number of CD11c^+^F4/80^+^, CD3^-^CD19^+^, and CD3^-^NK1.1^+^ cells infiltrating the LMMP was unaffected by HSV-1 infection (**Figure 4A** and data not shown). CD3^+^ cells increased significantly starting by the 6^th^ week of infection and the increase persisted up to the 10^th^ week post OG viral inoculation (**Figure 4B**).

To determine the distribution of the infiltrating CD3^+^ cells within the LMMP, ileal sections from the sham- and HSV-1-infected mice were probed with the anti-CD3 antibody. Immunostaining uncovered a negligible CD3^+^ infiltrate in the myenteric plexus and muscle layers of the sham infected mice and those sacrificed at the 4^th^ week of infection (**Figure 4C**). CD3^+^ cells surrounding the myenteric ganglia were evident 6 weeks post HSV-1 OG administration whereas CD3^+^ lymphocyte extensively infiltrated the myenteric ganglia 8 and 10 weeks post HSV-1 inoculum (**Figure 4C**). We further characterized the CD3^+^ infiltrate by flow cytometry analysis. CD3^+^CD4^+^ cells did not increase in the LMMP of HSV-1 infected mice as compared with sham infected animals (**Figure 4D**). On the contrary, the percentages of CD3^+^CD8^+^ and CD3^+^CD69^+^ (an early activation marker) cells infiltrating the LMMP were significantly higher at 6, 8, and 10 weeks post OG HSV-1 inoculum (**Figures 4E, F**).

### CD3^+^CD8^+^ Lymphocytes Infiltrating the LMMP Show an Activated Phenotype

**Figure 5:**
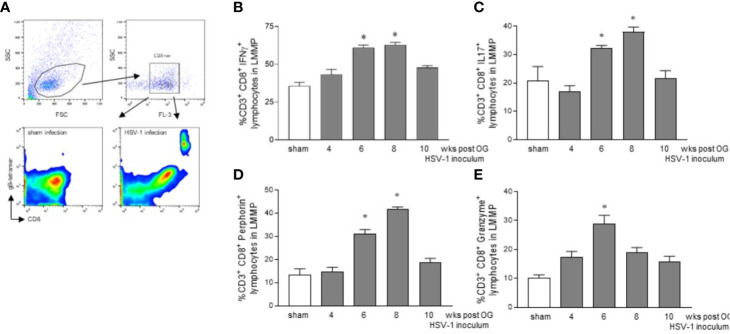
Activated phenotype in CD3^+^CD8^+^ lymphocytes infiltrating the LMMP. **(A)** The LMMP preparations of the ileum were obtained from sham and 8 wks OG HSV-1 infected mice. The samples were enzymatically digested. The resulting cell suspensions were incubated with anti-CD3, anti-CD8, and gB-tetramer and analyzed by flow cytometry. Gating strategy is reported in the first line: cells were first selected on a forward scatter (FSC) and side scatter (SSC) dot plot. CD3^+^ lymphocytes were then selected on FL-3/SSC dot plot. CD8 and gB-tetramer double positive cells were recorded in 10,000 events (second line). **(B–E)** The LMMP preparations were obtained from the ileum of the sham- and the HSV-1-infected mice. The samples were enzymatically digested, and the resulting cell suspensions were stained for intracellular cytokine analysis and analyzed by flow cytometry. Gating strategy is reported in **Figure S3**. Data report the percentage (mean±SEM) of CD8^+^ cells and IFNγ, IL17, perforin, or granzyme positive cells. * denotes *p *< 0.05 *vs* sham infected mice. Experiments were repeated 4 times n=4 mice per group.

To confirm the HSV-1 specificity of CD3^+^CD8^+^ cells identified *ex vivo*, LMMP preparations obtained from 8 wks HSV-1 infected mice (time at which we observed the higher HSV-1 gene expression, **Figure 1**) were enzymatically digested. Cell suspensions were stained with CD3, CD8, and gB-tetramers and analyzed by flow cytometry. Cells were first selected on a forward scatter (FSC) and side scatter (SSC) dot plot. CD3^+^ lymphocytes were then selected on an FL-3/SSC dot plot (**Figure 5A**). As reported in **Figure 5A**, we detected gB-CD8 positive cells in HSV-1 infected mice but not in sham infected mice.

Mononuclear cells isolated as described above from LMMP preparations of sham and HSV-1 infected mice were purified by density gradient centrifugation through Ficoll-Hypaque and stained for flow cytometric analysis. Cells were first selected in region 1 (R1) on a forward scatter (FSC) and side scatter (SSC) dot plot. CD3^+^ lymphocytes were selected in R1 on an FL-2/SSC dot plot (**Figure S3A**). For the intracellular cytokine analysis, only CD3^+^ cells were analyzed on FL-3 (CD8) and FL-1 (IFNγ, IL17, perforin, or granzyme) channels (**Figures 5**, **S3B–E**). We observed an increased expression of IFNγ in CD3^+^CD8^+^ lymphocytes infiltrating the LMMP of the mice 6 and 8 weeks post OG HSV-1 inoculum (**Figure 5B**). At the same time, CD8^+^ T cells in the LMMP expressed IL17, a cytokine involved in anti-viral response and the perpetuation of inflammation, particularly at the mucosal sites (Isailovic et al., 2015; Dunay et al., 2016). Finally, while the percentage of CD3^+^CD8^+^ cells expressing the pore-forming protein perforin was significantly higher 6 and 8 weeks post-IG HSV-1 inoculation, CD8^+^ lymphocytes expressing granzyme B increased at the 6^th^ week post OG inoculum (**Figures 5D, E**).

### T-Lymphocytes Infiltrating the LMMP Are Skewed Towards an HSV-1 Activated Phenotype

To determine whether the lymphocytes infiltrating the LMMP recognized the HSV-1 antigens, mononuclear cells isolated from the LMMP preparations of sham and HSV-1 infected mice were cultured and challenged *in vitro* with UV-inactivated HSV-1. The expression of IFNγ was assessed by intracellular cytokine staining and flow cytometry in CD3^+^CD8^+^ lymphocytes.

**Figure 6:**
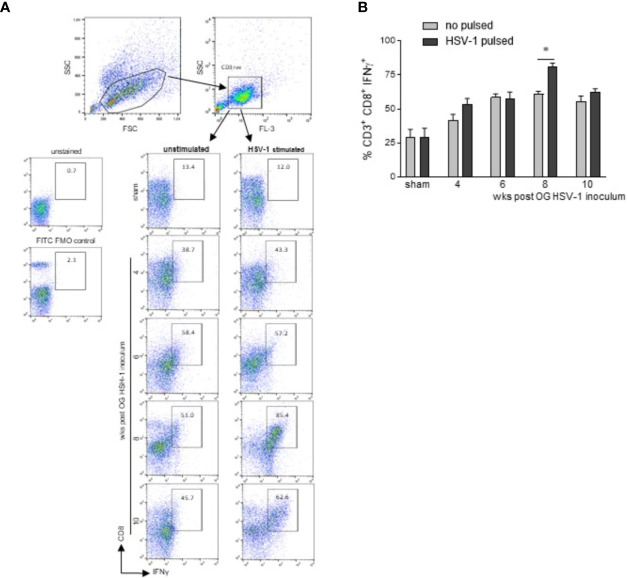
CD3^+^CD8^+^ lymphocytes infiltrating the LMMP are skewed towards HSV-1. Freshly collected LMMP were enzymatically digested and the resulting cell suspensions were incubated for 16 h in the presence or absence of UV-inactivated HSV-1. The cells were then collected, labeled with anti-CD3, anti-CD8, and anti-IFNγ antibodies for flow cytometric analysis. **(A)** Gating strategy is reported in the first line: cells were first selected on a forward scatter (FSC) and side scatter (SSC) dot plot. CD3^+^ lymphocytes were then selected on FL-3/SSC dot plot. CD8 and IFNγ double positive cells were recorded in 50,000 events in *in vitro* unstimulated or HSV-1 stimulated cells. The second line reports representative dot plots of 4 independent experiments; n=3 mice per experimental group. Unstained control and FITC (IFNγ) FMO control are also reported. **(B)** All data collected as described in **(A)** were graphed. Data are reported as the percentage (mean±SEM) of CD3^+^CD8^+^IFNγ^+^ cells in unstimulated or HSV-1 stimulated cultures. Experiments were repeated 4 times; n=3 mice per experimental group. * denotes *p*< 0.05 *vs* cells not incubated with UV-inactivated HSV-1 at the same time point.

Cells were first selected on a forward scatter (FSC) and side scatter (SSC) dot plot. CD3^+^ lymphocytes were then selected on an FL-3/SSC dot plot (**Figure 6A**). For the intracellular cytokine analysis, only CD3^+^ cells were analyzed on FL-2 (CD8) and FL-1 (IFNγ) channels in cells *in vitro* unstimulated or stimulated with HSV-1 previously inactivated by exposure to UV (**Figure 6A**). Following *in vitro* HSV-1 stimulation, a significant increase in IFN γ production was detected only in CD3^+^CD8^+^ cells cultured from mice at the 8^th^ week of infection (**Figure 6B**, *p*<0.05 *vs* cells not incubated with UV-inactivated HSV-1 at the same time point).

Activated cells infiltrating the LMMP expressed a specific chemokine pattern. Indeed, in the LMMP of the mice sacrificed at the 6^th^ week of infection, we identified increased levels of *Cxcl 11* mRNA specific transcripts (**Figure S4**), a key immune chemoattractant soluble factor involved in interferon-induced inflammatory responses (Kuo et al., 2018). Overall, our data indicate that HSV-1 infection recruits in the LMMP highly HSV-1 reactive CD8^+^ lymphocytes.

### Infiltrating CD8^+^ Lymphocytes Determine Intestinal Dysfunction During HSV-1 Infection of the Enteric Nervous System

**Figure 7:**
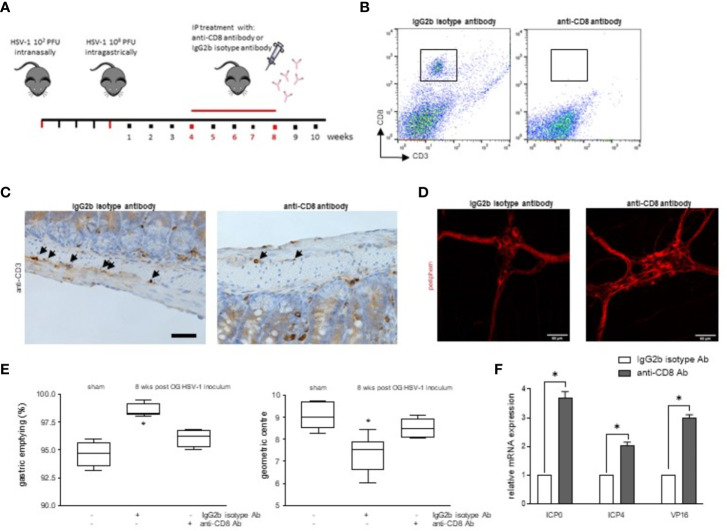
Depletion of CD8^+^ cells improved HSV-1 induced intestinal dysmotility. **(A)** Experimental design: at the 4^th^ week post HSV-1 OG administration, the mice were intraperitoneally injected with the anti-CD8 monoclonal antibody or IgG2b isotype antibody. The mice were sacrificed 4 weeks later. **(B)** LMMP preparations were obtained from the ileum of HSV-1-infected mice treated with anti-CD8 monoclonal antibody or IgG2b isotype antibody. LMMP were enzymatically digested, and the resulting cell suspensions were labeled with anti-CD3 and anti-CD8 antibodies and analyzed using flow cytometry. Cells were first selected on a forward scatter and side scatter dot plot and then double positive cells were recorded in 10,000 events. Representative images of 3 independent experiments; n=6 mice per group. **(C)** Sections of ileum collected from HSV-1 infected mice treated with anti-CD8 monoclonal antibody or IgG2b isotype antibody were subjected to immunohistochemistry for CD3. Representative images of 3 independent experiments. Representative images of 3 independent experiments. n=6 mice per experimental group; 4 independent fields per animal were examined. Scale bar: 40 µm. **(D)** Immunofluorescence for peripherin on whole mount preparations of distal ileum prepared from HSV-1 infected mice treated with anti-CD8 monoclonal antibody or IgG2b isotype antibody. Representative images of three independent experiments. Representative images of 3 independent experiments. n=6 mice per experimental group; 6 independent fields per animal were examined. Scale bars: 60 µm. **(E)** Sham infected and 8 wks HSV-1 OG infected mice treated with IgG2b isotype antibody or anti-CD8 antibody were administered *via* gastric gavage with non-absorbable FITC-labeled dextran and sacrificed 60 minutes later. Gastric emptying was calculated as the percentage of dextran retained in the stomach compared to the total fluorescence in the gastrointestinal tract. Intestinal transit refers to the geometric center that is the center of the distribution of fluorescent dextran in the ileum. Experiments were repeated 3 times; n=6 mice per group. * denotes *p* < 0.05 *vs* sham infected mice. **(F)** Total RNA was extracted from the LMMP of mice at the 8^th^ wks from the OG HSV-1 inoculum and treated with IgG2b isotype antibody or anti-CD8 antibody. Quantitative PCR was performed to evaluate the expression of HSV-1 genes. The data are reported as the mean ± SEM of gene levels detected in the mice treated with anti-CD8 antibody with respect to those identified in the infected animals dosed with IgG2b isotype antibody. Experiments were repeated 3 times; n=6 mice per group. * denotes *p* < 0.05 *vs* 8wks HSV-1 OG inoculum treated with IgG2b isotype antibody.

To investigate the pathophysiologic relevance on intestinal dysmotility (**Figure 2**) of CD3^+^CD8^+^ cells infiltrating the LMMP during HSV-1 infection of the ENS, four weeks after HSV-1 OG inoculum, mice were administered with monoclonal anti-CD8 antibody to deplete the specific T cell subset. The mice were sacrificed at the 8^th^ week of infection, the time at which we observed the higher HSV-1 tk expression (**Figure 7A**). Flow cytometric analysis demonstrated that the treatment with the anti-CD8 monoclonal antibody decreased intestinal CD3^+^CD8^+^ cells (**Figure 7B**). Moreover, the CD3^+^ cells surrounding the myenteric ganglia were significantly depleted in the infected mice administered with anti-CD8 antibody compared with mice dosed with IgG2b isotype antibody (**Figure 7C**).

The depletion of CD3^+^CD8^+^ cells ameliorated the HSV-1 induced neuronal damage and intestinal dysmotility in the HSV-1 infected mice. Indeed, expression of peripherin increased in anti-CD8 treated mice (**Figure 7D**). The gastric emptying and the geometric centre values were partially normalized, reaching levels comparable to sham infected mice (**Figure 7E**).

In neuronal ganglia, HSV-1 specific CD8 lymphocytes are actively involved in immunosurveillance against the virus, preventing reactivation or controlling replication (St. Leger and Hendricks, 2011). In our animal model, depletion of CD8^+^ cells in the LMMP activates the viral transcription of ICP0, ICP4, and VP16 genes (**Figure 7F**) but not gB or gC (data not shown) as demonstrated by increased levels of mRNA specific transcripts in the LMMP of anti-CD8 treated mice but not in mice treated with isotype antibody.

**Figure 8:**
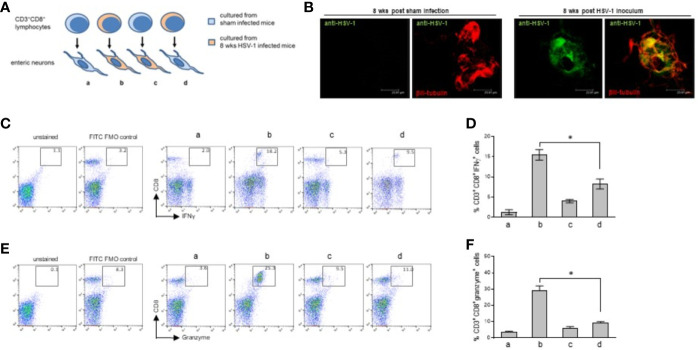
Immune interaction between infiltrating CD3^+^CD8^+^ lymphocytes and enteric neurons. **(A)** Experimental design: enteric neurons were isolated from sham or 8 weeks HSV-1 infected mice and cultured. Cells were co-incubated with CD3^+^CD8^+^ lymphocytes purified by immune sorting from cell suspensions obtained from the LMMP of the sham or HSV-1 infected mice. T cells isolated from sham infected mice were co-incubated with the enteric neurons cultured from sham **(a)** or HSV-1 infected **(c)** mice. T cells isolated from HSV-1 infected mice were co-incubated with enteric neurons cultured from the sham **(d)** or HSV-1 infected **(b)** mice. Twenty-four hours later, the lymphocytes were collected, labeled with anti-CD3, anti-CD8, anti-IFNγ, and anti-Granzyme antibodies, and analyzed by flow cytometry. **(B)** Enteric neurons isolated from the LMMP of sham or HSV-1 infected mice were cultured in the presence of acyclovir (100 μM) for nine days (see **Figure S5**). Acyclovir was removed and cells were cultured for additional 24 hours. Cells were then fixed and immunofluorescence for pan HSV-1 antigens (green) and βIII-tubulin (red) was performed. Representative images of 24 independent experiments; n=1 mouse per experimental group. Three independent fields per animal were examined. Scale bars: 23.81 µm. **(C, E)** T cells co-cultured with neurons were stained as described above and analyzed by flow cytometry. The gating strategy is the same described in **Figure S3**. CD8^+^IFNγ^+^ **(C)** and CD8^+^granzyme^+^ **(E)** cells (50,000 events) were analyzed in CD3^+^ cells. The experimental groups are denoted as a, b, c, d, as explained in **(A)**. Representative dot plots of 6 independent experiments; n=1 mouse per experimental group. Unstained control and FITC (IFNγ or granzyme) FMO control are also reported. **(D, F)** Data from all the experiments were graphed. Data report the percentage (mean±SEM) of **(D)** CD8^+^IFNγ^+^ cells in CD3^+^ gated cells and **(F)** CD8^+^Granzyme^+^ cells in CD3^+^ gated cells. The experimental groups in histograms are denoted as a, b, c, d, as explained in **(A)**. Experiments were repeated 6 times; n=1 mouse per experimental group. * denotes *p* < 0.05 *vs* **(C, D)**.

Co-culture experiments were designed to investigate further the role of the CD3^+^CD8^+^ lymphocytes in neuronal damage. Enteric neurons were isolated and cultured from the sham and 8 weeks HSV-1 infected mice and co-incubated with CD3^+^CD8^+^ lymphocytes sorted from cell suspensions obtained from the LMMP of the two groups of animals (**Figure 8A**). Enteric neurons were cultured for ten days, the time at which cells show well-organized ganglia and prolonged neurites (Brun et al., 2019). Acyclovir was added to cell culture to suppress lytic replication of the virus *in vitro*. Following the protocol described by Kobayashi et al., on the fifth day in culture we removed acyclovir from the culture media to allow viral antigen expression and we analyzed the cells for the expression of HSV-1 antigens and βIII-tubulin five days later (Kobayashi et al., 2012). As reported in **Figure S5A**, following five days in culture without acyclovir cells express HSV-1 antigens but βIII-tubulin positive cells appear smaller than neurons cultured from sham infected mice. Neurites were not detectable. To obtain suitable neuronal cultures, we removed acyclovir on the ninth day in culture and we analyzed cells 24 hours later. As reported in **Figure S5B**, HSV-1 antigens were detectable after 24 hours of acyclovir withdrawal and βIII-tubulin expression demonstrated well-organized ganglia and mature neurons. No specific HSV immunoreactivity was observed in neurons cultured for ten days with acyclovir. We therefore performed the next experiments in neurons cultured for 24 hours without acyclovir, the time at which immunoreactivity for the HSV-1 antigens was evident in βIII-tubulin^+^ neurons isolated from the HSV-1 infected but not from the sham-infected animals (**Figure 8B**).

Enteric neurons were co-cultured with CD3^+^CD8^+^ cells as described in **Figure 8A**. Twenty-four hours later we observed an increased expression of IFN-γ and granzyme in the lymphocytes isolated from the infected mice and co-cultured with *in vivo* infected neurons but not in the lymphocytes isolated from the LMMP of the HSV-1 infected mice and co-incubated with non-infected neurons (**Figures 8C–F**). The CD3^+^CD8^+^ lymphocytes isolated from the LMMP of the control mice and co-incubated with *in vivo* HSV-1 infected or sham-infected enteric neurons did not upregulate the expression of IFN-γ or granzyme (**Figures 8C–F**).

## Discussion

Infiltrating lymphocytes are suggestive of an immune-mediated mechanism and have been described in degenerative neuropathies that affect different districts of the body, including the ENS (De Giorgio et al., 2002; Törnblom et al., 2002; da Silveira et al., 2007; Veress et al., 2009). The events underlying and/or triggering pathophysiologic mechanism(s) of inflammation remain, however, largely unknown (De Giorgio et al., 2004; Dalakas, 2015). Compelling evidence supports the interaction between enteric neurons and inflammatory cells such as macrophages and mast cells (De Giorgio et al., 2004) and suggests that the neuropathies and gut motor disorders might result from a noxious insult (Collins et al., 1999; Spiller, 2002; de Lima et al., 2008). Our current study adds new insights in the field as we demonstrated that HSV-1 could infect and persist in the murine enteric neurons for up to 10 weeks following OG administration (**Figure 1**), damaging the myenteric ganglia (**Figure 3**) and resulting in intestinal dysmotility (**Figure 2**). In our animal model, the possible role played by the immune system is supported by the findings that enteric neurons cultured from *in vivo* HSV-1 infected mice express viral antigens (**Figure 8B**) and CD3^+^CD8^+^ cells responsive to HSV-1 antigens were retrieved from the myenteric plexus of the same animals (**Figure 6**). Since HSV-1 DNA has occasionally been found in the saliva of even asymptomatic subjects (Buddingh et al., 1954; Kaufman et al., 2005), these results could support the theories that in humans, HSV-1 particles might be swallowed during reactivation in the oral mucosa and might play a causative role in the inflammation of the enteric nervous system thus contributing to the onset of gastrointestinal dysfunctions. Several infective agents have been described to colonize the gut and subvert the tissue homeostasis leading to motor dysfunctions. In animal models, intestinal dysbiosis delays gastrointestinal transit through the involvement of the innate immune system (Anitha et al., 2016); in *Schistosoma mansomi*-infected mice the granulomatous inflammation correlates with increased muscle contractility and decreased gastrointestinal transit (Moreels et al., 2001); infection with influenza virus or *Salmonella* spp. increases intestinal paracellular permeability and ion transport (Worley et al., 2006; Sencio et al., 2021). In humans, a considerable number of bacteria, viruses, and parasites cause alterations in gastrointestinal motility and permeability or are involved in post-infectious inflammatory complications. Evidence supports the hypothesis that in addition to the direct effects on enterocytes *Cryptosporidium* spp., *Giardia* spp., cholera toxin, and Rotavirus evoke fluid, electrolyte secretion, and diarrhea by activation of the enteric nervous system (Spiller, 2002; Halliez and Buret, 2015). Neurotropic *Herpes* viruses have been demonstrated to replicate, persist and reactivate in the gastrointestinal tract (Chen et al., 2011; Gershon et al., 2012; Wang et al., 2015; Maidji et al., 2017), whereas HSV-1 has been retrieved in the nodose ganglia (Rand et al., 1984; Gesser and Koo, 1997; Gilden et al., 2001).

As previously reported, HSV-1 infection triggers chronic inflammation in the trigeminal ganglia (TG), and virus-specific T-cells play a pivotal role in controlling the HSV-1 lifecycle (van Velzen et al., 2013). At the same, in our study, we demonstrated that lymphocytes that infiltrate the myenteric plexus can control HSV-1 replication. At the 6^th^ and 8^th^ weeks after viral inoculum, T cells expressed higher levels of interferon, perforin, and granzyme (**Figure 5**), the mediators capable of inhibiting lytic gene expression (Kim et al., 2012). At the 6^th^ and 8^th^ weeks of infection, we also described fluctuating levels of HSV-1 gene expression, including LAT transcripts (**Figures 1A, B**). Thus, in our animal model, HSV-1 does not seem to shut down genome transcription completely during latency as elsewhere reported (Kramer and Coen, 1995; Feldman et al., 2002; Derfuss et al., 2007). We hypothesize that the immune responses can control only a subpopulation of enteric nerves *in vivo* (Liu et al., 2000), accounting for the simultaneous detection of latency, immediate early, and early genes, along with HSV-1 proteins (**Figure 1**). Nevertheless, when the CD8^+^ cells were depleted, we observed a significant increase in the levels of HSV-1 gene transcripts as compared with mice at the same time of infection (**Figure 7F**). We previously described fluctuating levels of HSV-1 specific transcripts and a temporally coordinated recruitment of macrophages at earlier times of infection, namely 1-3 weeks post OG viral inoculum (Brun et al., 2018; Brun et al., 2018). The immune response against the HSV-1 in the murine ENS appears highly coordinated and characterized by the activation of the innate immune system at the early time of infection when macrophages are recruited in the ENS by the CCL2/CCR2 pathway (Brun et al., 2018). In the current study, we reported that starting by the 4^th^ week of infection, the adaptive immune response driven by CD3^+^CD8^+^ cells (**Figure 4**) replaces the innate system and guides the intestinal alterations as confirmed by the partial amelioration of intestinal motility in mice in which CD8^+^ cells were depleted (**Figure 7E**). Indeed, it has been reported that both innate and adaptive immune responses and the related inflammatory cytokines block HSV-1 reactivation (Decman et al., 2005; Mott et al., 2014; Mott et al., 2016). The plasticity of the ENS reflects its ability to adapt to damages driven by the two immune system compartments and aims at maintaining the intestinal functions (Giaroni et al., 1999; Mawe et al., 2009). Neuroinflammation disturbs the excitability and synaptic properties of the enteric neurons and alters the neurochemical code of subpopulation of cells in the myenteric plexus (Lomax et al., 2017). Therefore, inflammatory stimuli and intestinal plasticity may have different functional consequences depending on the number and subtype of the involved neuronal cells (Lomax et al., 2005). In contrast, inflammation at one site of the gut wall can produce alterations at remotes sites in the gastrointestinal tract (Lomax et al., 2017). During the 1-10 weeks of HSV-1 infection, we observed structural, functional, and neurochemical alterations in the ENS (Brun et al., 2018) (**Figures 2**, **3**). HSV-1 related changes affect the whole gastrointestinal tract, from the stomach to the colon (**Figure 2**), with different effects. Indeed, we reported faster gastric emptying and delayed intestinal transit at the 2^nd^ and 8^th^ week of infection (Brun et al., 2018) (**Figure 2**). Deregulated intestinal motility have been described in different animal models and in patients suffering from inflammatory bowel diseases (Moreels et al., 2001; Hungin et al., 2005; Nóbrega et al., 2012). In our animal model, different neurochemical mediators and the involvement of cholinergic nerves need to be investigated. However, the results present in the current study prompt us to hypothesize that intestinal dysmotility results from the attempt of the ENS to counteract the inflammatory response elicited by the HSV-1 (Mawe et al., 2009).

In humans, T-cells infiltrating the TG recognize specific HSV-1 proteins and elicit an inflammatory response supported by *in situ* expression of cognate viral antigens that ensures continuous immune stimulation even in the absence of viral reactivation (Facco et al., 2008; van Velzen et al., 2013). In our model of HSV-1 infection of the ENS, we identified VP16 and gB protein expression in the myenteric plexus, but we were unable to detect gC and gD transcripts and gD protein (**Figure 1**). However, lymphocytes isolated from the ENS recognize HSV-1 gB (**Figure 5A**), are responsive to HSV-1 antigens (**Figure 6**) and are involved in gut dysmotility and neuronal damage (**Figures 7**, **8**). Neuronal damage is not usually described in HSV-1 infected TG, probably because, unlike enteric neurons, cranial nerves benefit from immune privilege and are protected from the potentially damaging effects of the inflammatory immune response (Theil et al., 2003). At the opposite, the intestinal lumen is enriched with antigenic potential that generates a pro-inflammatory milieu. In this setting, a limited number of infected neurons directly activate primed CD8^+^ T-cells or interact with antigen-presenting cells to amplify the anti-HSV-1 immune response through a cross-presentation process (Heath et al., 2001).

Our model of prolonged viral infection of the ENS in immunocompetent mice allowed us to unravel the kinetics of the immune response during a non-life-threatening HSV-1 infection. Indeed, activated CD8^+^ lymphocytes infiltrate the myenteric plexus starting by the 6^th^ week of infection and lasting until the 10^th^ week of infection (**Figures 4**, **5**). CD69, an early marker of lymphocyte activation, has been involved in TGF-β induction and suppression of IL-17 and IFN-γ production (Sancho et al., 2003; Radulovic et al., 2013). In our study, CD69 co-expressed with CD8^+^ T-cells that produce toxic mediators including IFN-γ, granzyme B, and perforin (**Figure 5**), suggesting that the anti-viral inflammatory response is partially blunted and a coordinated immune process is activated to avoid caspase-mediated killing of target cells and massive tissue damage. Indeed, CD3^+^ cells infiltrating the ENS lead to temporally restricted loss of integrity of intermediate filaments in neurons (**Figure 3**) with repeated fluctuations in functional neuromuscular alterations (**Figure 2**) as noted in pathological conditions characterized by neuronal sufferance (Liem and Messing, 2009; Tischfield et al., 2010). Remarkably, inflammatory-mediated abnormalities and CD3^+^ cells infiltrating the myenteric plexus are hallmarks of enteric nerve dysfunctions and gastrointestinal motor disorders, including achalasia, gastroparesis, chronic intestinal pseudo-obstruction, and slow-transit constipation (De Giorgio et al., 2004; Di Nardo et al., 2008).

In conclusion, our results lead us to hypothesize that the neuro-immune axis in the ENS is the checkpoint for the modulation of inflammation or tolerance against antigenic stimuli such as HSV-1 (Muller et al., 2014; Gabanyi et al., 2016). Indeed, infected neurons elicit the production of soluble factors by immune competent cells (Brun et al., 2018) whereas IFN-γ enhances the presentation of viral peptides, inhibits viral replication, and supports the Th1 response (Wing et al., 2008; Egan et al., 2013). Indeed, depletion of circulating CD8^+^ cells triggered the transcription of viral genes while the gastrointestinal transit was partially ameliorated (**Figure 7**). Previous studies showed that the disruption of CD8^+^ T-cells and the reduced capacity to secrete IFN-γ reactivates HSV-1 in the ganglionic neurons (90), whereas the plasticity of the ENS may counteract the new inflammatory milieu in the attempt to control intestinal functions (De Giorgio et al., 2004; Mawe et al., 2009). We think that these findings pose further questions about the role of inflammation in the neurophysiology of enteric microcircuits and the relevance in modulating IFN-γ levels as a therapeutic approach to gastrointestinal motor dysfunctions.

## Data Availability Statement

The original contributions presented in the study are included in the article and in **Supplementary Material**. Further inquiries can be directed to the corresponding author.

## Ethics Statement

The animal study was reviewed and approved by Animal Care and Use Committee of the University of Padova.

## Author Contributions

PB and IC designed the research, performed experiments, analyzed data, and drafted the manuscript; JC, VZ, VR, AK, and MeS performed experiments; AP, RC, MaS, MF, AC analyzed data, and critically read the manuscript. All authors contributed to the article and approved the submitted version.

## Funding

This research was funded by a grant to Ignazio Castagliuolo from the Italian Ministry of Education, Universities and Research (2009HLNNRL_002). The funding agencies played no role in designing the study protocol, nor in collecting/interpreting the data, nor in the decision to submit the manuscript for publication.

## Conflict of Interest

The authors declare that the research was conducted in the absence of any commercial or financial relationships that could be construed as a potential conflict of interest.

## Supplementary Material

Supplementary Figure 1 Histological analysis of the ileum in the HSV-1 infected mice. Sections of the ileum collected from the sham- and HSV-1-infected mice were stained with H&E. Representative images of 6-8 mice per group and at least 10 independent fields per animal were examined. Images were analyzed using a Leica microscope equipped with a digital camera. Scale bars: 40 µm.

Supplementary Figure 2 Gastrointestinal dysmotility in sham infected mice. Sham infected mice were OG dosed with non-absorbable FITC-labeled dextran and sacrificed 60 minutes later. **(A)** Gastric emptying was calculated as the percentage of dextran retained in the stomach with respect to the total amount of fluorescence in the gastrointestinal tract. **(B)** Intestinal transit refers to the geometric center that is the center of the distribution of fluorescent dextran in the ileum. Data are reported as mean ± SEM. The experiments were repeated 3 times; n=6 mice per group. As data in sham infected mice were comparable among the different points, data were pulled together and reported as a unique sham infected group in **Figure 2**.

Supplementary Figure 3 CD3^+^ cells infiltrate the LMMP following HSV-1 infection. The LMMP preparations obtained from the ileum of sham and HSV-1 infected mice were enzymatically digested, and the resulting cell suspensions were stained for intracellular cytokine analysis and analyzed by flow cytometry. **(A)** Gating strategy for data reported in **Figures 5B–E**. Cells were first selected on a forward scatter (FSC) and side scatter (SSC) dot plot. CD3^+^ lymphocytes were selected in R1 on an FL-2/SSC dot plot. **(B–E)** For the intracellular cytokine analysis, only CD3^+^ cells were analyzed on CD8^+^ cells and IFNγ, IL17, perforin, and granzyme positive cells (50,000 events). Representative dot plots of 4 independent experiments; n=4 mice per experimental group. Data obtained from all the experiments are reported in **Figures 5B–E**.

Supplementary Figure 4 Chemokine expression pattern in the LMMP of the infected mice. Quantitative RT-PCR analysis of *Ccl22* **(A)**, *Cxcl 11* **(B)**, *Cxcl 10* **(C)**, and *Cxcl9* **(D)** mRNA in the LMMP from the sham- and HSV-1-infected mice. The data were normalized to *Rn18S* expression levels and reported as fold change. The experiments were repeated three times; n=6 mice per group. * denotes *p*<0.05 *vs* sham infected mice.

Supplementary Figure 5 Set up protocol for culture of HSV-1 infected enteric neurons. Enteric neurons were isolated in cultured for 10 days from 8 wks HSV-1 infected mice or sham infected mice. At the time of cell seeding, acyclovir (100 μM) was added to cell culture to suppress lytic replication of the virus. To allow viral antigen expression, acyclovir was withdrawn at different times. Cells were then fixed and stained with anti pan HSV-1-FITC conjugated antibody (in green) and anti-βIII- tubulin-PE conjugated antibody (in red). **(A)** Acyclovir was removed on the 5^th^ day in culture and cells were cultured for additional 5 days. **(B)** Acyclovir was removed on the 9^th^ day in culture and cells were cultured for additional 24 hours. **(C)** Acyclovir was not removed and cells were cultured for 10 days in the presence of acyclovir. For each experimental condition, βIII-tubulin staining in neurons cultured from sham infected animals is reported. Representative images of two independent exoeriments; n=2 mice per experimental group. Scale bars: 75 µm.
