# Supplementary figures and images for: Persistent Herpes Simplex Virus Type 1 Infection of Enteric Neurons Triggers CD8+ T Cell Response and Gastrointestinal Neuromuscular Dysfunction
Source: Front Cell Infect Microbiol. 2021 May 18;11:615350. doi: 10.3389/fcimb.2021.615350 (PMC8169984; doi:10.3389/fcimb.2021.615350)

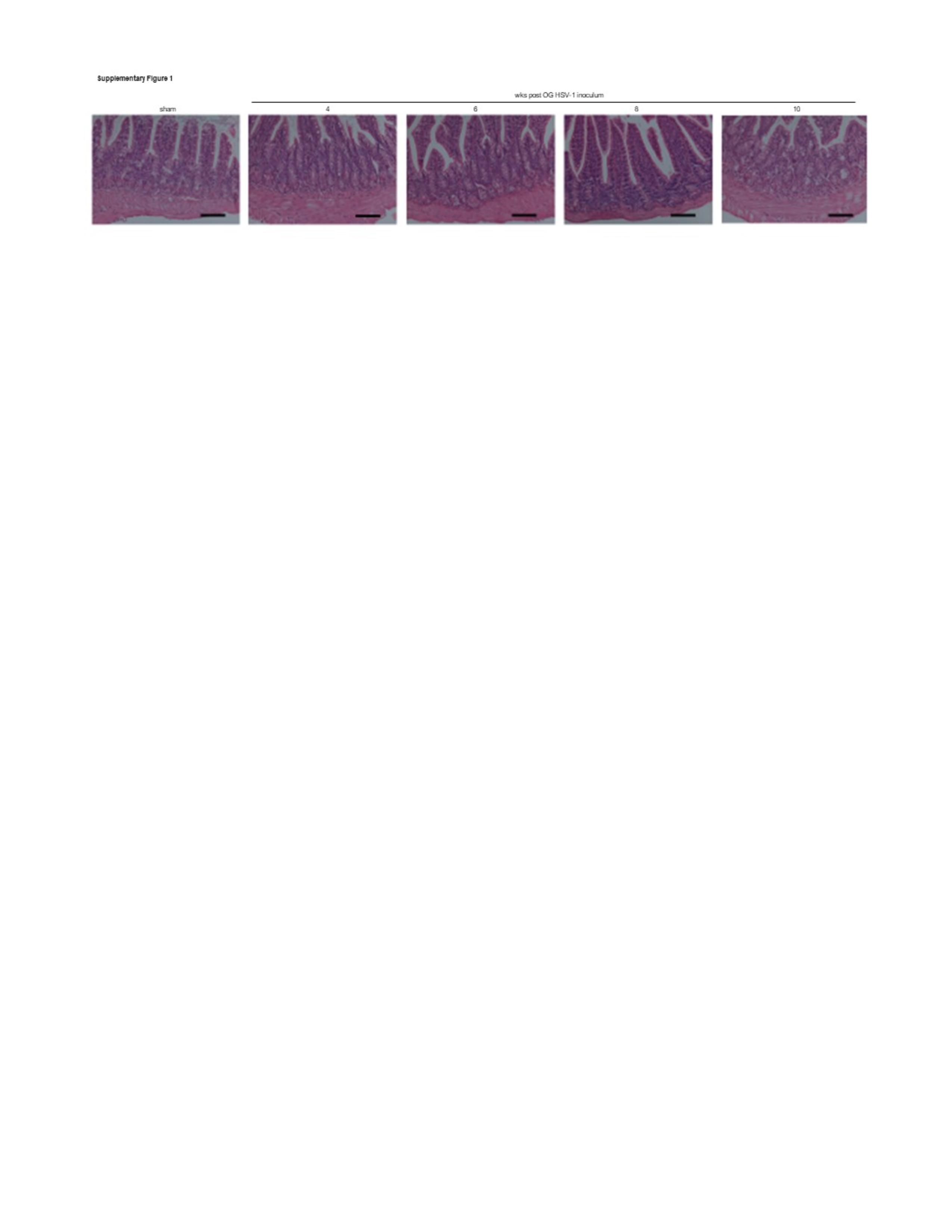

Supplement: Supplementary Figure 1 — Histological analysis of the ileum in the HSV-1 infected mice. Sections of the ileum collected from the sham- and HSV-1-infected mice were stained with H&E. Representative images of 6-8 mice per group and at least 10 independent fields per animal were examined. Images were analyzed using a Leica microscope equipped with a digital camera. Scale bars: 40 µm. [file Image_1.tif]

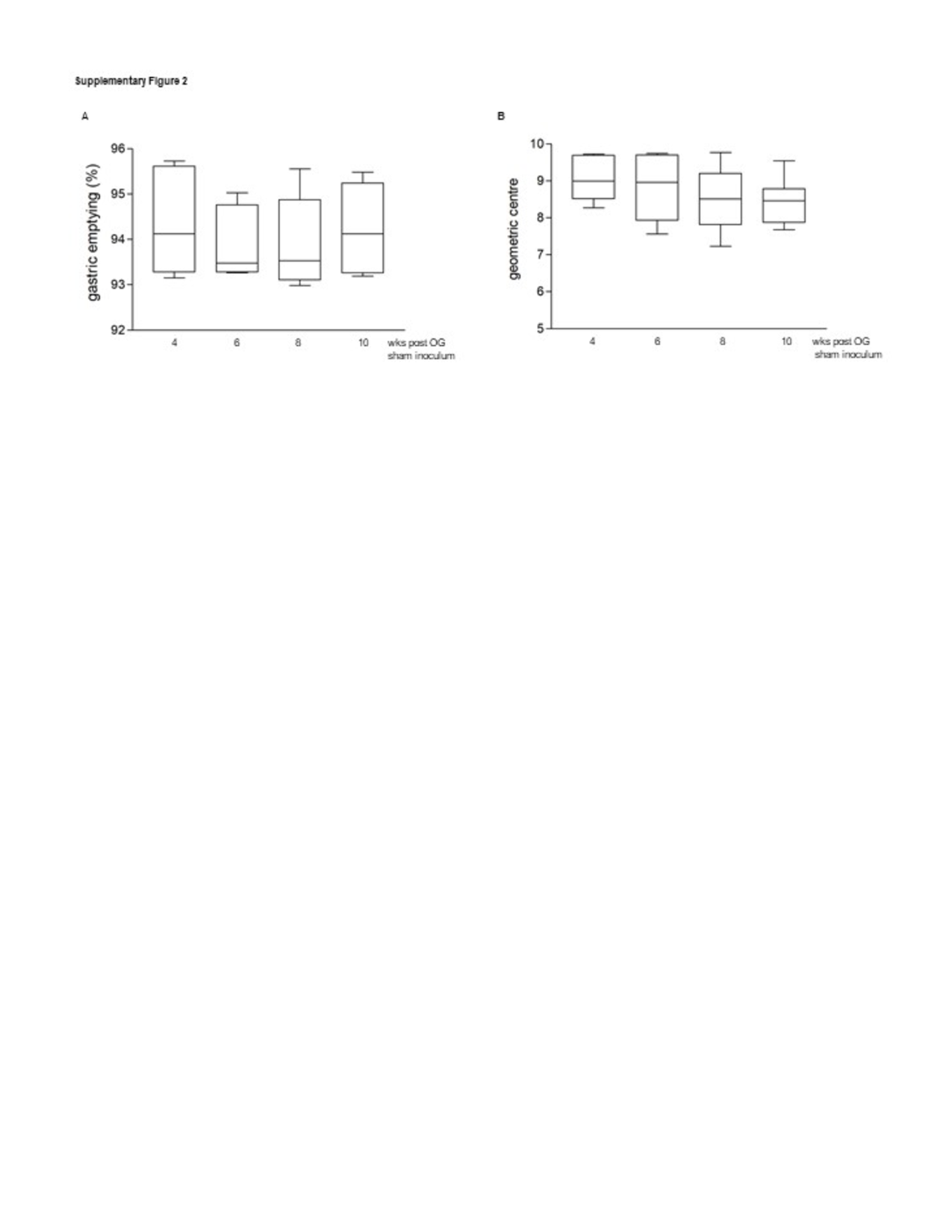

Supplement: Supplementary Figure 2 — Gastrointestinal dysmotility in sham infected mice. Sham infected mice were OG dosed with non-absorbable FITC-labeled dextran and sacrificed 60 minutes later. (A) Gastric emptying was calculated as the percentage of dextran retained in the stomach with respect to the total amount of fluorescence in the gastrointestinal tract. (B) Intestinal transit refers to the geometric center that is the center of the distribution of fluorescent dextran in the ileum. Data are reported as mean ± SEM. The experiments were repeated 3 times; n=6 mice per group. As data in sham infected mice were comparable among the different points, data were pulled together and reported as a unique sham infected group in Figure 2 . [file Image_2.tif]

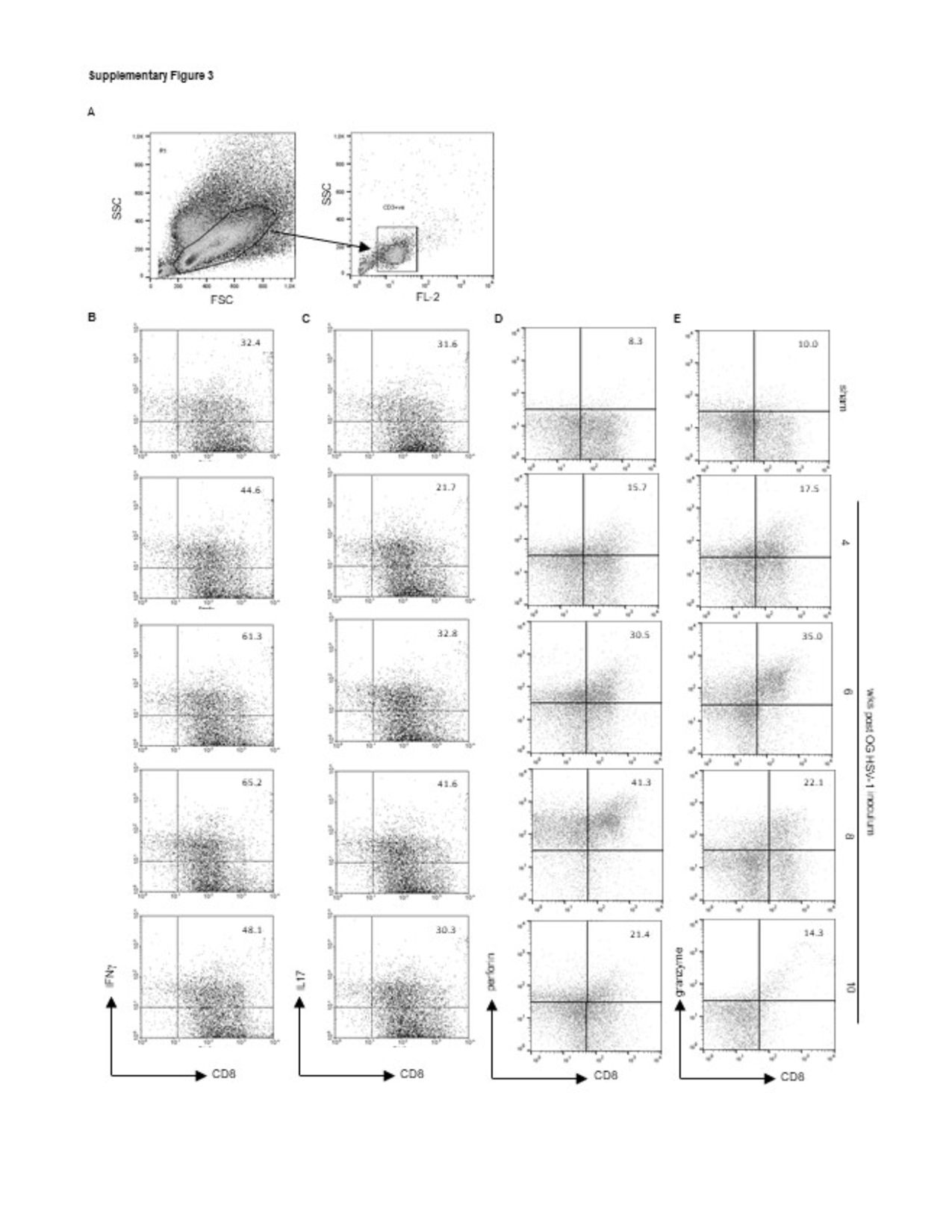

Supplement: Supplementary Figure 3 — CD3+ cells infiltrate the LMMP following HSV-1 infection. The LMMP preparations obtained from the ileum of sham and HSV-1 infected mice were enzymatically digested, and the resulting cell suspensions were stained for intracellular cytokine analysis and analyzed by flow cytometry. (A) Gating strategy for data reported in Figures 5B–E . Cells were first selected on a forward scatter (FSC) and side scatter (SSC) dot plot. CD3+ lymphocytes were selected in R1 on an FL-2/SSC dot plot. (B–E) For the intracellular cytokine analysis, only CD3+ cells were analyzed on CD8+ cells and IFNγ, IL17, perforin, and granzyme positive cells (50,000 events). Representative dot plots of 4 independent experiments; n=4 mice per experimental group. Data obtained from all the experiments are reported in Figures 5B–E . [file Image_3.tif]

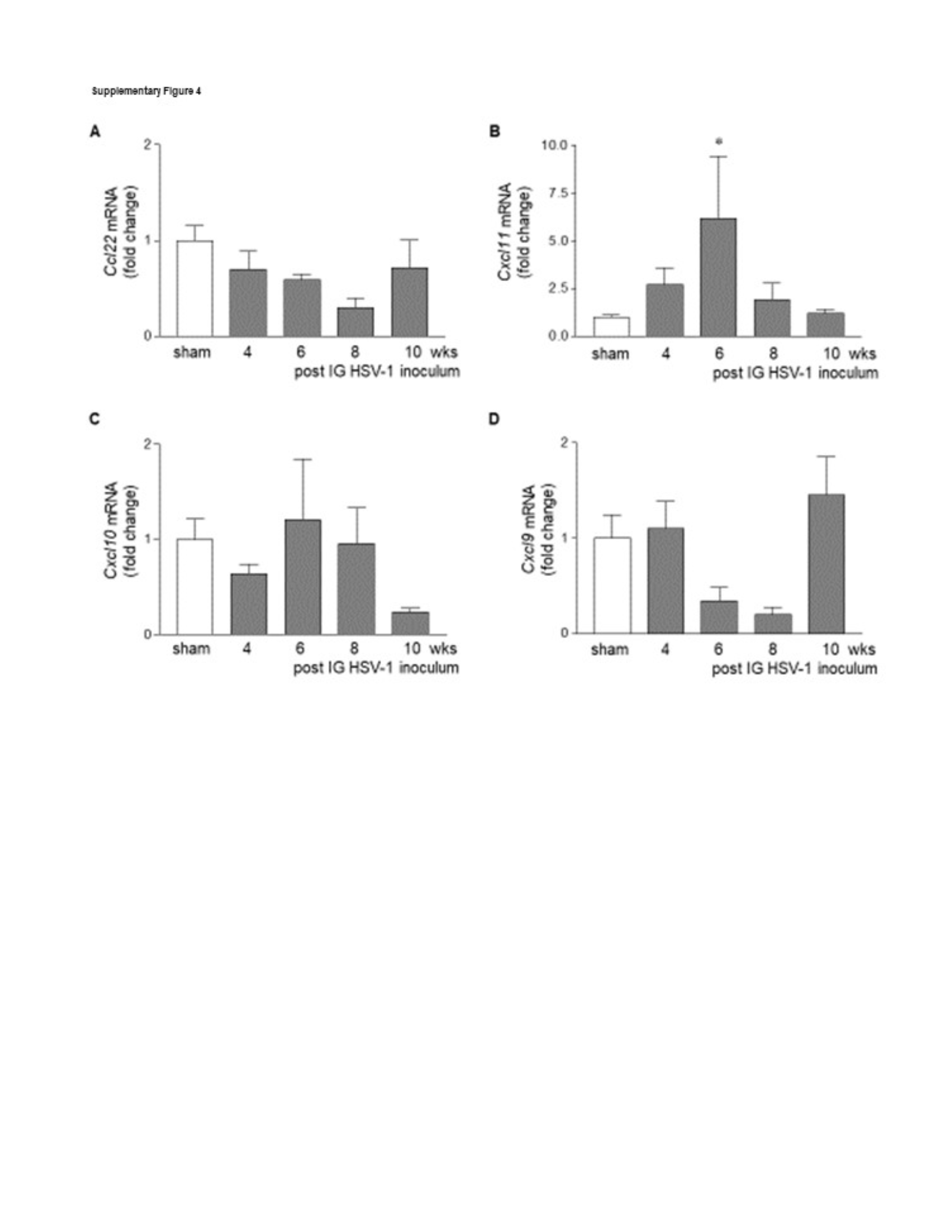

Supplement: Supplementary Figure 4 — Chemokine expression pattern in the LMMP of the infected mice. Quantitative RT-PCR analysis of Ccl22 (A), Cxcl 11 (B), Cxcl 10 (C), and Cxcl9 (D) mRNA in the LMMP from the sham- and HSV-1-infected mice. The data were normalized to Rn18S expression levels and reported as fold change. The experiments were repeated three times; n=6 mice per group. * denotes p<0.05 vs sham infected mice. [file Image_4.tif]

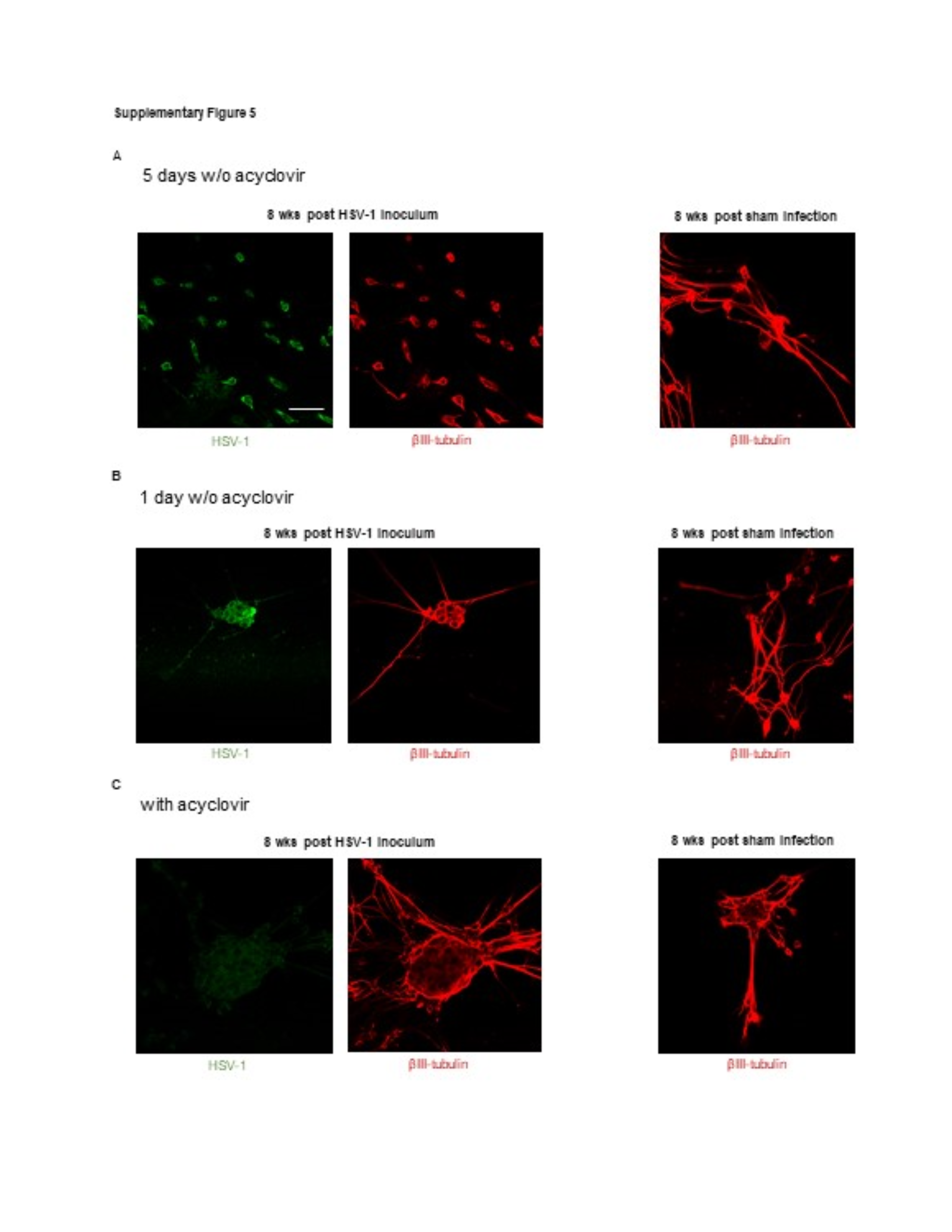

Supplement: Supplementary Figure 5 — Set up protocol for culture of HSV-1 infected enteric neurons. Enteric neurons were isolated in cultured for 10 days from 8 wks HSV-1 infected mice or sham infected mice. At the time of cell seeding, acyclovir (100 μM) was added to cell culture to suppress lytic replication of the virus. To allow viral antigen expression, acyclovir was withdrawn at different times. Cells were then fixed and stained with anti pan HSV-1-FITC conjugated antibody (in green) and anti-βIII- tubulin-PE conjugated antibody (in red). (A) Acyclovir was removed on the 5th day in culture and cells were cultured for additional 5 days. (B) Acyclovir was removed on the 9th day in culture and cells were cultured for additional 24 hours. (C) Acyclovir was not removed and cells were cultured for 10 days in the presence of acyclovir. For each experimental condition, βIII-tubulin staining in neurons cultured from sham infected animals is reported. Representative images of two independent exoeriments; n=2 mice per experimental group. Scale bars: 75 µm. [file Image_5.tif]
